# Evaluating the consequences of salmon nutrients for riparian organisms: Linking condition metrics to stable isotopes

**DOI:** 10.1002/ece3.2697

**Published:** 2017-02-01

**Authors:** Carmella Vizza, Beth L. Sanderson, Holly J. Coe, Dominic T. Chaloner

**Affiliations:** ^1^ National Marine Fisheries ServiceNorthwest Fisheries Science CenterSeattleWAUSA; ^2^ Department of Biological SciencesUniversity of Notre DameNotre DameINUSA

**Keywords:** aquatic–terrestrial subsidies, body condition index, marine‐derived nutrients, mixing models, Pacific salmon

## Abstract

Stable isotope ratios (δ^13^C and δ^15^N) have been used extensively to trace nutrients from Pacific salmon, but salmon transfer more than carbon and nitrogen to stream ecosystems, such as phosphorus, minerals, proteins, and lipids. To examine the importance of these nutrients, metrics other than isotopes need to be considered, particularly when so few studies have made direct links between these nutrients and how they affect riparian organisms. Our study specifically examined δ^13^C and δ^15^N of riparian organisms from salmon and non‐salmon streams in Idaho, USA, at different distances from the streams, and examined whether the quality of riparian plants and the body condition of invertebrates varied with access to these nutrients. Overall, quality and condition metrics did not mirror stable isotope patterns. Most notably, all riparian organisms exhibited elevated δ^15^N in salmon streams, but also with proximity to both stream types suggesting that both salmon and landscape factors may affect δ^15^N. The amount of nitrogen incorporated from Pacific salmon was low for all organisms (<20%) and did not correlate with measures of quality or condition, probably due to elevated δ^15^N at salmon streams reflecting historical salmon runs instead of current contributions. Salmon runs in these Idaho streams have been declining, and associated riparian ecosystems have probably seen about a 90% reduction in salmon‐derived nitrogen since the 1950s. In addition, our results support those of other studies that have cautioned that inferences from natural abundance isotope data, particularly in conjunction with mixing models for salmon‐derived nutrient percentage estimates, may be confounded by biogeochemical transformations of nitrogen, physiological processes, and even historical legacies of nitrogen sources. Critically, studies should move beyond simply describing isotopic patterns to focusing on the consequences of salmon‐derived nutrients by quantifying the condition and fitness of organisms putatively using those resources.

## 1 Introduction

The nutrients that Pacific salmon (*Oncorhynchus* spp.) bring to freshwater streams influence adjacent riparian and terrestrial ecosystems (Helfield & Naiman, 2001; Naiman, Bilby, Schindler, & Helfield, 2002), which are often traced through the food web using stable isotopes of carbon and nitrogen. There are several pathways by which salmon‐derived nutrients (SDN) can be transferred to terrestrial ecosystems including: (1) floods depositing salmon carcasses on stream banks (Cederholm, Houston, Cole, & Scarlett, 1989), (2) subsurface flows transferring dissolved nutrients excreted by salmon to riparian areas (O'Keefe & Edwards, 2002), (3) terrestrial predators and scavengers moving salmon carcasses away from the stream and distributing SDN in the form of urine and feces (Ben‐David, Hanley, & Schell, 1998; Hilderbrand, Hanley, Robbins, & Schwartz, 1999), and (4) emergent aquatic insects acting as a minor vector for SDN to riparian habitats (Francis, Schindler, & Moore, 2006). Riparian organisms can incorporate SDN through these different pathways, thereby influencing the isotopic patterns observed in their tissues. For example, riparian vegetation only uses nitrogen from salmon because plants fix carbon from the atmosphere (Ben‐David et al., 1998). In contrast, invertebrates can obtain salmon‐derived carbon and nitrogen by directly consuming salmon carcasses, and can also indirectly acquire salmon‐derived nitrogen from isotopically enriched vegetation and herbivorous consumers (Hocking & Reimchen, 2002). Through these pathways, riparian plants and invertebrates alongside streams receiving salmon spawners often exhibit higher δ^15^N, but not necessarily higher δ^13^C (Ben‐David et al., 1998; Bilby, Beach, Fransen, Walter, & Peter, 2003; Helfield & Naiman, 2001).

Many studies have used changes in carbon and nitrogen stable isotope ratios to infer the importance of salmon resources to recipient ecosystems. These studies usually involve estimating the percentage of an organism's diet composed of salmon using mixing models (Bilby, Fransen, & Bisson, 1996; Kline, Goering, Mathisen, Poe, & Parker, 1990), but to truly establish the importance of salmon resource subsidies, we need other physiological metrics to complement stable isotopes of carbon and nitrogen (e.g., Rinella, Wipfli, Walker, Stricker, & Heintz, 2013). This is especially important because salmon contribute other nutrients that cannot necessarily be traced with stable isotopes, such as phosphorus, minerals, amino acids, fats, carbohydrates, and other essential organic compounds (Olsen, 1999). The availability of these nutrients could be just as vital to producers and consumers as the carbon and nitrogen alone (Wipfli, Hudson, Caouette, & Chaloner, 2003). Additionally, Gannes, O'Brien, and del Rio (1997) argued that stable isotope ratios can be correlated with dietary, trophic level, and body condition patterns, which means that correctly interpreting the importance of SDN requires some understanding of physiological processes. Unfortunately, few terrestrial SDN studies have established a direct relationship between changes in stable isotope ratios and physiological metrics of organisms (but see Hilderbrand, Jenkins, Schwartz, Hanley, & Robbins, 1999; Tonra, Sager‐Fradkin, & Marra, 2016), whereas this practice has been more common in aquatic studies with fish as the recipient organisms (e.g., Bilby, Fransen, Bisson, & Walter, 1998; Kiffney, Buhle, Naman, Pess, & Klett, 2014; Rinella, Wipfli, Stricker, Heintz, & Rinella, 2011; Swain, Hocking, Harding, & Reynolds, 2013). Direct consumption of salmon resources could enhance the body condition of organisms via increased fat storage (Hilderbrand, Jenkins, et al. 1999), or indirectly increase condition by providing a higher‐quality resource including nutrients that limit growth (Chaloner & Wipfli, 2002; Minakawa, Gara, & Honea, 2002; Wipfli et al., 2003). Establishing this link is crucial in determining whether there are longer‐term ecological consequences of SDN, such as for survivorship and reproduction of recipient organisms.

To better understand how SDN benefits organisms, some measure of fitness is needed. Because an organism's fitness (i.e., lifetime or reproductive success) can be challenging to measure, ecologists will often measure “condition,” which is a snapshot of an organism's physiological state (Jakob, Marshall, & Uetz, 1996). For vegetation, one measurement generated routinely during stable isotope analysis is the C:N ratio. This ratio reveals the relative nitrogen content of the foliage, with low C:N ratios generally indicating a faster turnover in primary producers and greater rates of herbivory (Cebrián, Williams, McClelland, & Valiela, 1998). Together, stable isotope and C:N ratios could indicate whether vegetation is of higher quality because of uptake of salmon‐derived nitrogen (Helfield & Naiman, 2001). Similarly to plants, consumer C:N ratios can be indicators of food quality for the next trophic level (Elser et al., 2000), but consumer C:N ratios can also be correlated with lipid content, a relationship that is not consistently found in plants (Post et al., 2007). The influence of SDN on consumer C:N ratio is dependent on which salmon‐derived elements are utilized by consumers. If consumers were primarily utilizing nitrogen from salmon, then lower C:N ratios would be expected, whereas if they were incorporating salmon‐derived carbon from lipids and fatty acids, one might expect higher C:N ratios. Due to this complexity, we therefore chose to complement C:N ratios with use of a body condition index, a commonly used metric for invertebrate consumers (Jakob et al., 1996; Kotiaho, 1999; Moya‐Laraño, Macías‐Ordóñez, Blanckenhorn, & Fernández‐Montraveta, 2008; Uetz, Papke, & Kilinc, 2002). Body condition indices use individual length and mass as a proxy of the energy reserves established in the body (Peig & Green, 2009), and thus reveal information about an organism's ability to survive and reproduce.

The main goal of our study was to determine whether the quality or condition of terrestrial organisms is affected by SDN. To establish the longer‐term influence of SDN on riparian food webs, we compared plant and invertebrates from salmon (S) and non‐salmon streams (NS), with varying proximity from the stream channel (i.e., 0 and 100 m). Our first objective was to determine how the riparian invertebrates chosen in this study incorporate SDN. If invertebrates were directly consuming carcasses, we expect that δ^13^C and δ^15^N of these organisms would be higher at salmon streams (S > NS) and that direct consumption would decline farther away from the channel (S_0_ > S_100_). However, if invertebrates were incorporating SDN indirectly, we would expect that only δ^15^N would follow those patterns (S > NS, S_0_ > S_100_). Secondly, we wanted to determine how SDN influenced riparian vegetation quality or invertebrate body condition. We hypothesized that vegetation C:N ratio would be lower (i.e., higher quality) along salmon streams (S < NS), and would increase with distance from salmon streams (S_0_ < S_100_), reflecting carcass availability. Similarly, we hypothesized that invertebrate body condition at salmon streams would be higher (S > NS) and that proximity to the channel was only important adjacent to salmon streams (S_0_ > S_100_, NS_0_ = NS_100_). Finally, we wanted to determine whether we could directly link the individual condition of riparian plants and invertebrates at salmon streams to the percentage of nitrogen in their tissues that was salmon‐derived. We hypothesized that riparian plant quality and invertebrate condition would increase with our estimates of salmon‐derived nitrogen. By establishing the effect that SDN have on recipient populations, this study has broader implications for studies attempting to link ecology with physiology and for restoration projects with the goal of re‐instating community dynamics prior to declining salmon runs.

## 2 Materials and methods

### 2.1 Study area

**Figure 1:**
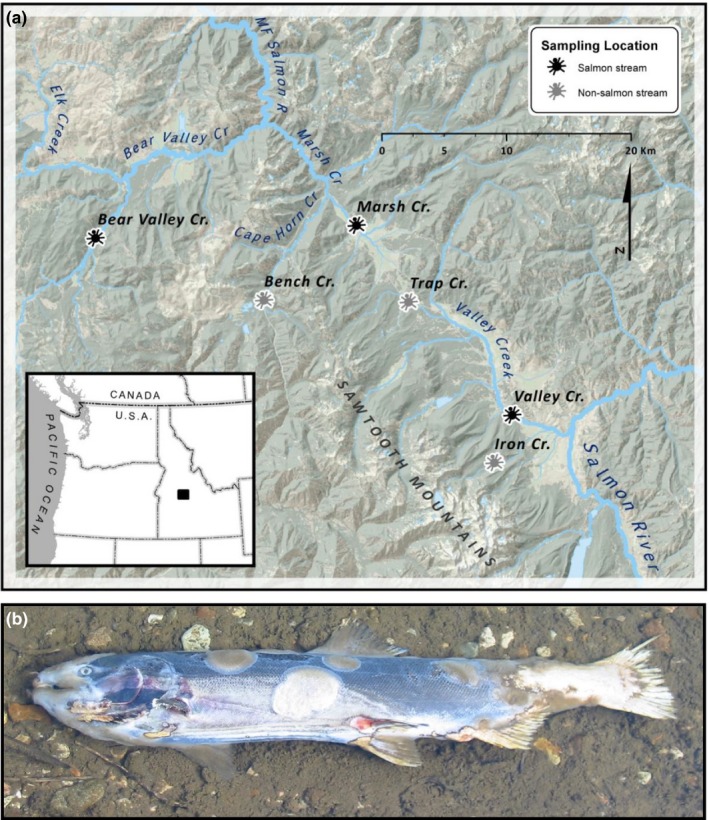
(a) Location of the three salmon streams (black symbol) and three non‐salmon streams (grey symbol) sampled in the Salmon River watershed, Idaho, USA. (b) Picture of a Chinook salmon carcass (*Oncorhynchus tshawytscha*) in an Idaho stream

The study was conducted in three salmon and three non‐salmon streams in the Salmon River Basin in central Idaho, USA, within the U.S. National Forest System (Figure 1). All six streams are located within the Level III Idaho Batholith ecoregion, whose bedrock geology consists of Cretaceous granitic rocks (McGrath et al., 2002). Salmon and non‐salmon streams are geomorphically similar, but non‐salmon streams are impassable to Pacific salmon due to physical barriers which impede the upstream migration of adult fish. Salmon streams support Chinook salmon (*Oncorhynchus tshawytscha*), rainbow trout/steelhead (*O. mykiss*), bull trout (*Salvelinus confluentus*), non‐native brook trout (*S. fontinalis*), and sculpin (*Cottus* spp.); non‐salmon tributaries are dominated by brook trout and sculpin (Sanderson, Tran, et al. 2009). The forested landscape is dominated by Ponderosa pine (*Pinus ponderosa*) and Douglas fir (*Pseudotsuga menziesii*) with riparian vegetation consisting of mostly sedges (*Carex* spp.) and willow (*Salix* spp.). All six of the streams were low‐gradient systems with low to moderate canopy cover (Table 1).

**Table 1:** Study area information for the six streams sampled during summer 2009, including stream type (S, salmon and NS, non‐salmon). The gradient or slope refers to the stream channel, and cover percentages are averages from three transects per stream

Stream	Type	National Forest	Elevation (m)	Discharge (m^3 ^s^−1^)^a^	Slope %	Riparian plot	Ground cover %	Mid‐layer cover %	Canopy cover %
Bench Creek (BEN)	NS	Salmon‐Challis	2,110	0.15	0.56	0 m	95	23	32
						100 m	88	10	23
Bear Valley Creek (BVA)	S	Boise	1,985	1.40	0.26	0 m	71	30	20
						100 m	58	41	56
Iron Creek (IRO)	NS	Sawtooth	2,039	0.42	2.39	0 m	49	73	50
						100 m	88	31	70
Marsh Creek (MAR)	S	Salmon‐Challis	2,003	1.41	0.81	0 m	67	41	0
						100 m	64	42	5
Trap Creek (TRA)	NS	Sawtooth	2,030	0.24	0.01	0 m	58	49	15
						100 m	70	17	61
Valley Creek (VAL)	S	Sawtooth	1,925	2.94	0.55	0 m	43	65	0
						100 m	95	28	0

a Discharge measurements were collected in July 2010 at one transect per stream.

### 2.2 Vegetation and invertebrate collection

Sampling was conducted during 2009 when spawners were present. Three riparian sampling transects were chosen per stream. Transects were spaced 200 m apart and extended 100 m perpendicular from the channel edge. For each transect, we characterized the vegetation in two 10 m × 10 m plots, at the water's edge (0 m) and 100 m away from the channel. Ground cover and mid‐layer percentages of vegetation were quantified using visual estimates, and canopy cover was measured using a densiometer (Kaufmann, Levine, Robison, Seelinger, & Peck, 1999; Table 1). We chose to collect *Carex* spp. because they were an abundant herbaceous plant found at both the water's edge and 100 m from the channel. Foliage samples were kept on ice for no longer than 1 week due to the remote nature of the sites and then frozen at –20^○^C for long‐term storage.

Using the same plots, we collected riparian invertebrates that could consume either aquatic invertebrates emerging from the streams or terrestrial invertebrates that fed on riparian vegetation, which are both potential vectors of SDN. Formicidae (ants) and Araneae (spiders) were collected with forceps or aspirators and then placed into jars with 80% ethanol. Ethanol preservation can alter δ^13^C (Kaehler & Pakhomov, 2001; Sarakinos, Johnson, & Zanden, 2002; Vizza, Sanderson, Burrows, & Coe, 2013), but this was critical to ensure that these predators died instantly instead of preying on each other. No correction of δ^13^C was necessary as all invertebrates were treated the same way, and because we only used δ^13^C of the invertebrates in this study to confirm that there was no direct consumption of salmon carcasses. We believe that our selection of riparian plants and invertebrates is appropriate because these organisms grow rapidly and have lifespans lasting a few years, and therefore, their stable isotope ratios are most likely to reflect the presence of SDN accumulated in their tissue either currently or within the last few salmon runs. In addition, the consumers we chose were of smaller size, which allowed us to sample the entire organism instead of having to subsample specific tissues.

### 2.3 Invertebrate identification and measurement

Formicidae and Araneae were identified to genus, the lowest possible taxonomic level, using Fisher and Cover (2007) and Ubick, Paquin, Cushing, and Roth (2005). The most predominant genera (*Formica* spp. for ants and *Pardosa* spp. for wolf spiders) were selected to compare similar taxa across streams. Lower taxonomic resolution was not necessary as the species in each genus were morphologically similar and because all members of the *Formica* genus are omnivores and those of the *Pardosa* genus are functionally carnivores (Fisher & Cover, 2007; Ubick et al., 2005). Because *Formica* spp. differ morphologically by social caste, and *Pardosa* spp. exhibit extreme sexual dimorphism, we standardized selection of specimens by ensuring that *Formica* spp. were from a worker caste and *Pardosa* spp. were females. These selection criteria were implemented to minimize any possible caste or sex bias that could affect body condition metrics, but also resulted in different sample sizes across locations (Table 2). After selection, we measured head capsule width for *Formica* spp. and total body length (head to abdomen) for *Pardosa* spp., which were the best predictors of body size by taxa. Measurements were made to the nearest 0.5 μm using an ocular micrometer mounted on a dissecting microscope at 10× magnification. The invertebrates were then dried at 50^○^C for 24 hr and weighed to the nearest 0.001 mg (*Formica* spp.) or 0.1 mg (*Pardosa* spp.).

**Table 2:** Sample size (*n*) for each stream and distance (0 and 100 m) grouping of ants (*Formica* spp.) and wolf spiders (*Pardosa* spp.) summed across three transects. Stream type (S, salmon and NS, non‐salmon) is also indicated

Stream	Type	Distance	*Formica*	*Pardosa*
Bench Creek	NS	0 m	42	52
		100 m	36	32
Bear Valley Creek	S	0 m	44	24
		100 m	49	13
Iron Creek	NS	0 m	24	17
		100 m	42	22
Marsh Creek	S	0 m	53	61
		100 m	50	19
Trap Creek	NS	0 m	36	30
		100 m	32	21
Valley Creek	S	0 m	36	50
		100 m	52	17

### 2.4 Body condition index

Body condition indices are commonly used in laboratory and field studies with Araneae (Jakob et al., 1996; Moya‐Laraño et al., 2008; Uetz et al., 2002), but this metric has not been widely used in *Formica* spp. Determining the condition of insects with different social castes is undoubtedly a challenge, and one could argue that colony size might be the best indicator of fitness or condition. However, there is no apparent correlation between climate and colony size, or even colony size and its longevity (Hölldobler & Wilson, 1990). Targeting queens for fitness or condition estimates would have been ideal except we were unable to collect enough of this caste. Instead, we chose to focus on workers because of their abundance and the role they play in rearing reproductive females (Hölldobler & Wilson, 1990). The ability of a worker to carry out this role is often linked to the special nutrients it produces in the exocrine glands, and there is a strong positive correlation between the secretion of these fertile substances and the storage of lipids in *Formica* workers (Hölldobler & Wilson, 1990). Because lipid content is highly correlated with C:N ratios in animals (Post et al., 2007), one might expect that workers in better condition would have a higher lipid content and C:N ratio. To validate the use of a body condition metric in *Formica* spp., we therefore compared our index to the C:N ratio of these *Formica* spp. and found that these two metrics were positively correlated (r = .68, *df* = 188, *p *<* *.001).

Specifically, the body condition of each invertebrate was estimated using a scaled mass index (Peig & Green, 2009):

$$\mathrm{Scaled}\;\mathrm{mass}\;\mathrm{index}\;(\mathrm{SMI})=M_{i}{\left[L_{0}/L_{i}\right]}^{b}$$

where *M*_*i*_ is body mass and *L*_*i*_ is a length measurement of individual *i*,* L*_*0*_ is the mean body size for the study population, and *b* is the scaling exponent. The scaling exponent is the slope from a standard major axis regression of ln‐transformed observations of mass versus length. The scaled mass index allows different‐sized invertebrates to be compared by predicting the mass of an individual for the standard body size (*L*_*0*_). Many different methods exist for estimating body condition (Jakob et al., 1996), but we chose SMI for the following reasons (after Peig & Green, 2009, 2010): (1) this index accounts for the changing relationship between mass and length as body size changes and growth occurs via the scaling exponent, thus allowing for a valid comparison between individuals of a different size; (2) there is error involved in measuring both length and mass, and so this method uses a standardized major axis regression that takes into account error in both the *x* and *y* variables; and (3) SMI is a more reliable indicator of body composition than other indices (Peig & Green, 2009).

### 2.5 Stable isotope analysis

All samples were freeze‐dried for 24 hr, and then pulverized using scissors to minimize sample loss. While *Pardosa* spp. were large enough to analyze individually, we pooled *Formica* spp. in groups of two to three individuals to generate sufficient material for analyses. Approximately 1.8–2.0 mg (vegetation) or 0.5–0.7 mg (invertebrates) of homogenized powder was weighed into tin capsules for analysis. Stable isotope ratios were determined using a Costech ECS 4010 elemental analyzer coupled to a Thermo Electron Delta Plus stable isotope ratio mass spectrometer as previously described in Sanderson, Tran, et al. (2009). Reported values have a precision of at least 0.3‰ for δ^15^N and 0.2‰ for δ^13^C, and ratios of C:N were determined from percent element data (% C and % N).

### 2.6 Percentage of salmon‐derived nitrogen incorporated

To estimate the percentage of salmon‐derived nitrogen being incorporated into the riparian plants and invertebrates, we used a two‐source mixing model (after Francis et al., 2006):

$$\delta^{15}N_{S0}=P_{\mathrm{salmon}}\left(\delta^{15}N_{\mathrm{salmon}}+\beta L\right)+\left(1-P_{\mathrm{salmon}}\right)\delta^{15}N_{S100}$$

where δ^15^N_*S0*_ is the δ^15^N for the plant or invertebrate taxon collected from a salmon stream at 0 m, *P*_salmon_ is the proportion of plant or invertebrate nitrogen derived from salmon, δ^15^N_salmon_ is the δ^15^N of Chinook salmon carcass tissue (14.2‰; B. Sanderson, unpublished data), β is the average enrichment between trophic levels (3.4‰; Post, 2002), *L* is the number of trophic levels between the consumer and the salmon, and δ^15^N_*S100*_ is the corresponding average δ^15^N for the plant or invertebrate taxa collected from the same stream at 100 m. With the assumption that salmon input 100 m away from the stream was negligible as no evidence of carcasses was present, we used δ^15^N_*S100*_ as the baseline instead of the corresponding δ^15^N of a taxon at non‐salmon streams because it was the most conservative (i.e., δ^15^N_*S100*_ > δ^15^N_*NS0*_). In estimating the percentage of salmon‐derived nitrogen in this manner, we made a simplifying assumption that the only difference in nitrogen uptake between organisms at 0 and 100 m away from the stream was the presence of salmon nutrients. While this assumption is essential to the mixing model, it could lead to underestimates of salmon‐derived nitrogen if salmon influence extends more than 100 m beyond the stream. This assumption could also lead to an overestimate of salmon‐derived nitrogen if the main riparian source of nitrogen tended to be higher in δ^15^N closer to the stream rather than 100 m away for reasons other than salmon influence. Another key assumption included that riparian plants accumulate salmon‐derived nitrogen directly from uptake through the soil (*L *=* *0), because they are not consumers (i.e., fractionation of N isotopes occurs with excretion; Gannes, del Rio, & Koch, 1998). Although *Formica* spp. can be omnivorous, we assumed that their role was primarily as secondary consumers because they were observed gathering predominantly insects, not vegetation. Because Francis et al. (2006) showed that aquatic insects play a minor role in dispersing SDN into riparian forests, we assumed that riparian invertebrates consumed herbivorous insects that fed on riparian vegetation that came in contact with salmon carcasses in order to acquire SDN (*L *=* *2). This assumption was also supported by riparian invertebrate δ^13^C. These values would have been higher, especially at the water's edge, if invertebrates obtained SDN more directly via carcasses or organisms that consume carcasses. Then we solved this equation for *P*_salmon_ and converted it to a percentage representing the amount of salmon‐derived nitrogen incorporated into each taxon at the salmon streams.

### 2.7 Statistical analyses

We conducted all analyses of variance (ANOVAs, α = 0.05) using a split‐plot design (Gotelli & Ellison, 2004):

$$Y_{ijk}=\mu +A_{i}+B_{j(i)}+C_{k}+AC_{ik}+CB_{k(i)}\left[+\varepsilon_{ijkl}\right],$$

where the whole‐plot treatment (*A*_*i*_) is salmon or nonsalmon, the different plots nested within *A* are the streams (*B*_*j*(*i*)_), the within‐plot factor (*C*_*k*_) is the distance from stream (0 and 100 m), and the error term is ε_*ijkl*_. To test for the effect of salmon *A*, we used the mean square for the streams *B* as denominator for the *F*‐ratio (Gotelli & Ellison, 2004). The within‐plot treatment of distance *C* and the interaction of salmon and distance *A *× *C* were both tested against the stream–distance interaction *B *× *C* term (Gotelli & Ellison, 2004). We conducted these analyses of variance with the following response variables: (1) δ^13^C for *Formica* spp., (2) δ^13^C for *Pardosa* spp., (3) δ^15^N for *Carex* spp., (4) δ^15^N for *Formica* spp., (5) δ^15^N for *Pardosa* spp., (6) C:N ratio for *Carex* spp., (7) C:N ratio for *Formica* spp., (8) C:N ratio for *Pardosa* spp., (9) SMI for *Formica* spp., and (10) SMI for *Pardosa* spp. Stable isotope ratios (δ^13^C and δ^15^N) met the assumptions of ANOVA so no transformations were necessary (Zar, 2010), whereas both C:N ratio and SMI were transformed using natural logarithms to address violations of normality (Zar, 2010). To evaluate whether the percentage of salmon‐derived nitrogen incorporated into the riparian taxa at salmon streams affects their quality (C:N ratio) or body condition (SMI), we ran separate correlations for *Carex* spp., *Formica* spp., and *Pardosa* spp. If the percentage of salmon‐derived nitrogen was found to be zero, those individuals were removed from the correlation analyses. Salmon‐derived nitrogen percentages, C:N ratios, and SMI were transformed using natural logarithms to address violations of normality (Zar, 2010).

## 3 Results

### 3.1 Stable isotope ratios

Riparian invertebrate δ^13^C was similar or even lower at salmon streams than at non‐salmon streams. *Formica* δ^13^C did not differ between non‐salmon and salmon streams (NS: −24.6‰ ± 0.2‰, S: −24.7‰ ± 0.2‰, *F*_1,4_ = 0.25, *p *=* *.64), but tended to increase farther away from the stream (0 m: −24.8‰ ± 0.3‰, 100 m: −24.5‰ ± 0.3‰, *F*_1,4_ = 4.80, *p *=* *.09). In contrast, *Pardosa* δ^13^C was lower at salmon streams (NS: −24.9‰ ± 0.6‰, S: −26.0‰ ± 0.1‰, *F*_1,4_ = 15.79, *p *=* *.02), and increased farther from the stream (0 m: −25.7‰ ± 0.6‰, 100 m: −24.9‰ ± 0.7‰, *F*_1,4_ = 31.63, *p *=* *.005).

**Figure 2:**
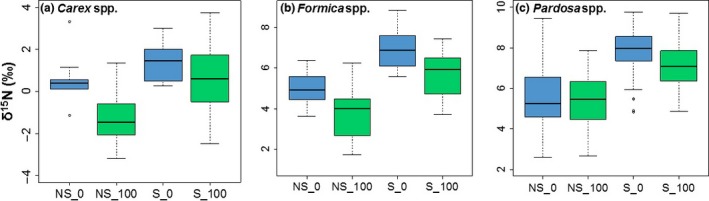
Box plots of δ^15^N for (a) sedges (*Carex* spp.), (b) ants (*Formica* spp.), and (c) spiders (*Pardosa* spp.) separated by stream type (NS, non‐salmon; S, salmon) and distance from the channel, with 0 m in blue and 100 m in green. Whiskers mark the lowest datum still within 1.5 of the interquartile range of the lower quartile and the highest datum still within 1.5 of the interquartile range of the upper quartile

**Figure 3:**
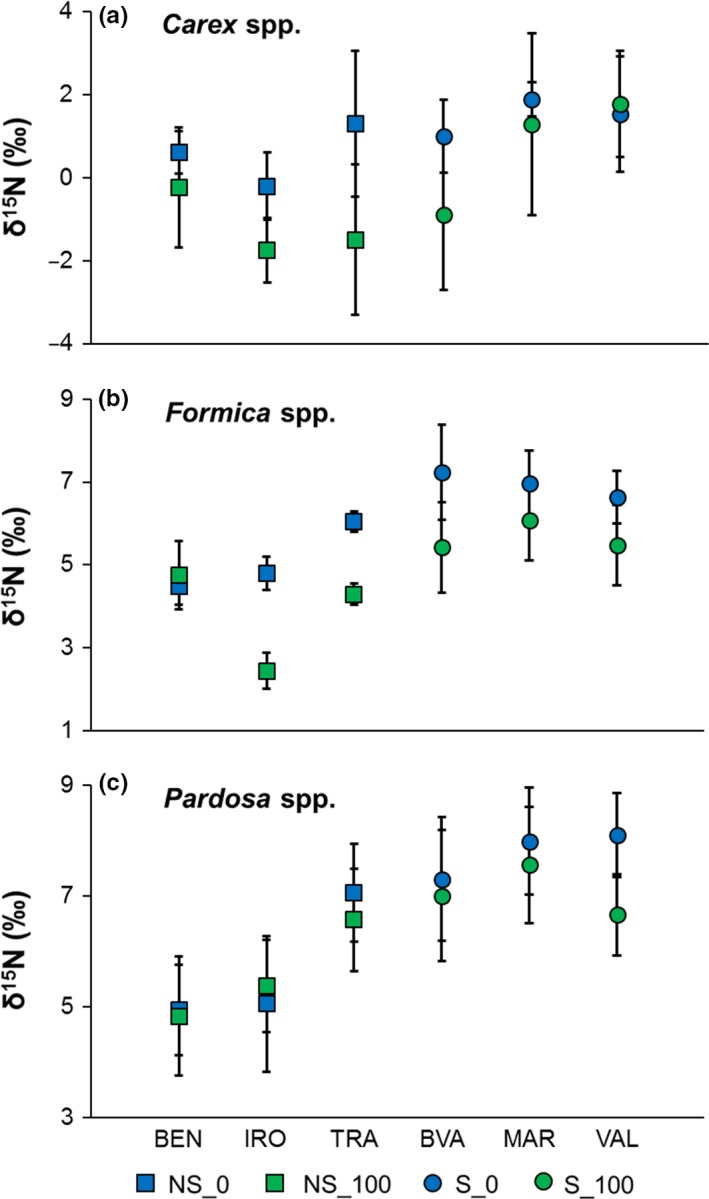
Mean ± *SD* δ^15^N for (a) sedges (*Carex* spp.), (b) ants (*Formica* spp.), and (c) spiders (*Pardosa* spp.) broken down by each individual stream. Distance from the channel is indicated with color whereby 0 m is blue and 100 m is green. Non‐salmon streams are represented by squares and salmon streams are represented by circles

Riparian plant and invertebrate δ^15^N were higher adjacent to salmon streams than non‐salmon streams (Figure 2). *Carex* δ^15^N tended to be higher at salmon streams (*F*_1,4_ = 4.91, *p *=* *.09) and was significantly lower at 100 m from both stream types (*F*_1,4_ = 8.62, *p *=* *.05, Figure 2a). Additionally, *Formica* δ^15^N was significantly higher at salmon streams (*F*_1,4_ = 16.71, *p *=* *.02) and at 0 m (*F*_1,4_ = 8.97, *p *=* *.04, Figure 2b), and *Pardosa* δ^15^N was significantly higher at salmon streams (*F*_1,4_ = 11.99, *p *=* *.03) and tended to be higher at 0 m (*F*_1,4_ = 4.41, *p *=* *.10, Figure 2c). The effect of distance on δ^15^N did not change between salmon and non‐salmon streams (interaction: *p *>* *.10 for all taxa), but the magnitude of the change did appear to vary across individual streams (Figure 3).

### 3.2 C:N ratios and invertebrate body condition

**Figure 4:**
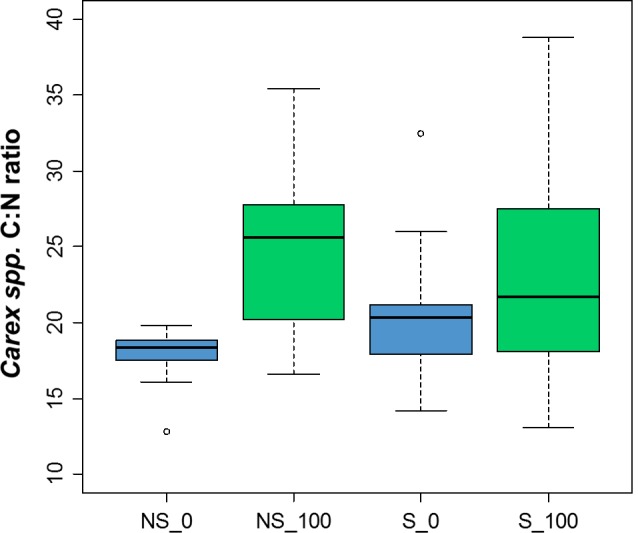
Box plot of sedge (*Carex* spp.) C:N ratio separated by stream type (NS, non‐salmon; S, salmon) and distance from the channel, with 0 m in blue and 100 m in green. Whiskers mark the lowest datum still within 1.5 of the interquartile range of the lower quartile and the highest datum still within 1.5 of the interquartile range of the upper quartile

**Figure 5:**
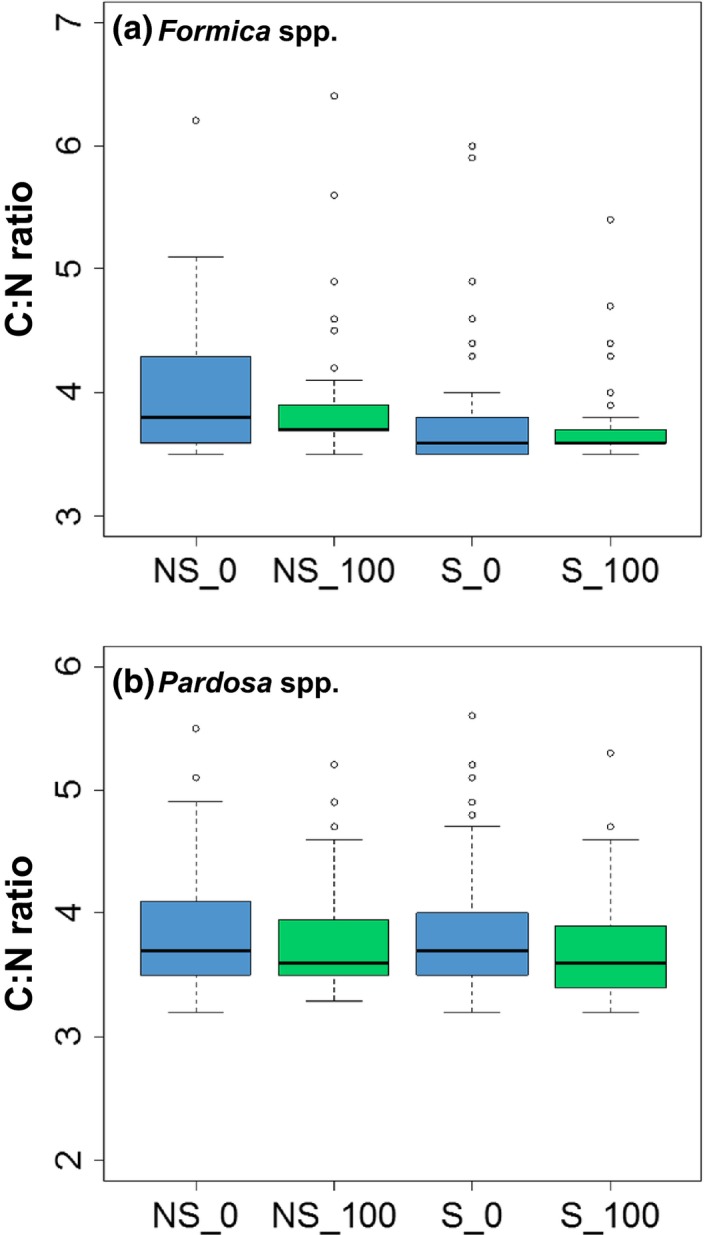
Box plots of C:N ratios for (a) ants (*Formica* spp.) and (b) spiders (*Pardosa* spp.) separated by stream type (NS, non‐salmon; S, salmon) and distance from the channel, with 0 m in blue and 100 m in green. Whiskers mark the lowest datum still within 1.5 of the interquartile range of the lower quartile and the highest datum still within 1.5 of the interquartile range of the upper quartile

**Figure 6:**
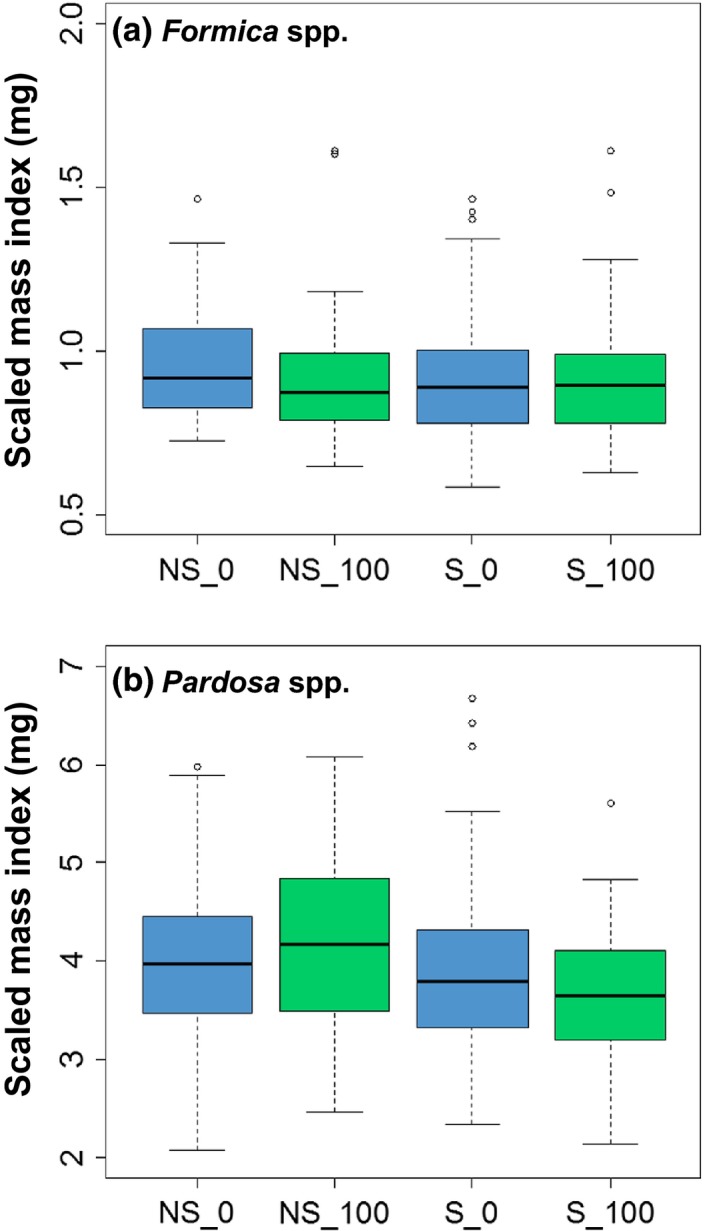
Box plots of body condition indices (scaled mass index) for (a) ants (*Formica* spp.) and (b) spiders (*Pardosa* spp.) separated by stream type (NS, non‐salmon; S, salmon) and distance from the channel, with 0 m in blue and 100 m in green. Whiskers mark the lowest datum still within 1.5 of the interquartile range of the lower quartile and the highest datum still within 1.5 of the interquartile range of the upper quartile

Nutritional quality of plants and body condition of riparian invertebrates did not differ between salmon and non‐salmon streams. The C:N ratio of *Carex* spp. did not vary with stream type (*F*_1,4_ = 0.15, *p *=* *.72), but was significantly lower at 0 m than at 100 m (*F*_1,4_ = 8.07, *p *=* *.05, Figure 4). In contrast, neither stream type (*Formica*:* F*_1,4_ = 1.75, *p *=* *.26, Figure 5a; *Pardosa*:* F*_1,4_ = 0.08, *p *=* *.79, Figure 5b) nor proximity to the channel (*Formica*:* F*_1,4_ = 1.29, *p *=* *.32, Figure 5a; *Pardosa*:* F*_1,4_ = 3.25, *p *=* *.15, Figure 5b) affected the C:N ratio of either invertebrate group. Similarly, there was no effect of stream type (*F*_1,4_ = 0.03, *p *=* *.87) or proximity to the channel on *Formica* SMI (*F*_1,4_ = 1.23, *p *=* *.33, Figure 6a), and *Pardosa* SMI did not differ by stream type (*F*_1,4_ = 1.60, *p *=* *.27) or with distance from the stream (*F*_1,4_ = 0.13, *p *=* *.74, Figure 6b). No interaction between stream type and distance was observed for any taxa (*p *>* *.10 for all taxa).

### 3.3 Percentage of salmon‐derived nitrogen incorporated

The amount of salmon‐derived nitrogen incorporated into the tissues of riparian taxa was low (<20%). According to mixing model calculations, *Carex* spp. near salmon streams incorporated about 9.8% ± 9.0% of nitrogen from salmon. There was no correlation between salmon‐derived nitrogen estimates in plant tissue and the C:N ratios of the plants (r = −.12, *df* = 5, *p *=* *.79). *Formica* spp. accumulated 9.7% ± 6.5% of their nitrogen from salmon, whereas *Pardosa* spp. accumulated 7.8% ± 6.2% of their nitrogen from salmon. Our estimates of salmon‐derived nitrogen were weakly, but positively, correlated with C:N ratio for *Formica* spp. (r = .31, *df* = 46, *p *=* *.03), but they were not correlated with *Pardosa* spp. (r = .03, *df* = 108, *p *=* *.75). Additionally, salmon‐derived nitrogen estimates were not correlated with SMI for either *Formica* spp. (r = −.01, *df* = 46, *p *=* *.94) or *Pardosa* spp. (r = −.06, *df* = 108, *p *=* *.51).

## 4 Discussion

Salmon resources influence recipient ecosystems via a variety of mechanisms. To date, such mechanisms include increased ambient nutrient concentrations (Gende, Miller, & Hood, 2007; Mitchell & Lamberti, 2005), altered ecosystem metabolism (Holtgrieve & Schindler, 2010), increased growth and densities of consumers (Chaloner & Wipfli, 2002; Gende & Willson, 2001), and enhanced growth rate or other element of individual fitness (Hilderbrand, Jenkins, et al. 1999; Swain et al., 2013). This study examined the last mechanism and found very little evidence that the current contribution of salmon resources—inferred using stable isotopes and mixing models—increased the quality of riparian vegetation or the condition of terrestrial invertebrates. Several plausible explanations discussed below have implications for future understanding of the influence of salmon resource subsidies on ecosystems and for the broader use of stable isotopes in ecological research.

### 4.1 Stable isotope ratios

Isotopic evidence suggests that invertebrates were accumulating SDN via indirect riparian pathways (e.g., consumption of herbivorous insects that feed on vegetation such as *Carex* spp.). If *Formica* and *Pardosa* individuals were consuming either salmon carcass material directly, or necrophagous organisms that feed on carcass material, we would expect an increase in *Formica* and *Pardosa* δ^13^C and δ^15^N at salmon streams (Hocking & Reimchen, 2002). However, *Formica* and *Pardosa* δ^13^C were either the same or lower than at non‐salmon streams, which suggests that invertebrates adjacent to these streams primarily derive their carbon from the riparian base of the food web. Nonetheless, the elevated δ^15^N of riparian plants and invertebrates at salmon streams indicates that when SDN are assimilated, they are taken up and incorporated indirectly from the riparian soil pool. Riparian plant communities, which include *Carex* spp., play a crucial role in transferring energy and nutrients from the riparian soil pool up to higher trophic levels when consumed by herbivorous insects. Specifically, these plants can accumulate SDN via subsurface flows or transfer of salmon carcasses to land, but we also know that plant type can moderate the effect of salmon on δ^15^N (Ben‐David et al., 1998). For example, Ben‐David et al. (1998) found that soil fertilization was likely unimportant for skunk cabbage (*Lysichiton americanus*) with its localized nutrient cycling and high nitrogen levels. In contrast, the patterns of δ^15^N in *Carex* spp. reflected some importance of salmon‐derived nitrogen for this riparian plant. Nonetheless, our observation that δ^15^N of *Carex* was also higher closer to non‐salmon streams suggest that factors in addition to salmon are at play.

Both salmon and landscape factors likely influenced riparian plant and invertebrate δ^15^N. We observed higher δ^15^N of all taxa at salmon streams than non‐salmon streams, which is consistent with previous studies that have also found elevated δ^15^N in riparian vegetation of salmon streams (Bartz & Naiman, 2005; Ben‐David et al., 1998; Bilby et al., 2003; Helfield & Naiman, 2001). We also found that δ^15^N decreased 100 m from both stream types, suggesting that salmon alone cannot explain δ^15^N patterns. Denitrification is a reasonable explanation for elevated δ^15^N close to both stream types (Pinay, O'Keefe, Edwards, & Naiman, 2003) because this process frequently takes place in the floodplain of low‐gradient streams where soils are often water‐logged and experience anaerobic conditions (Pinay et al., 2007). Denitrification rates are also likely to be higher at streams with elevated nitrate concentrations (Mulholland et al., 2008), but all of the streams in this study had low nitrate levels (<25 μg N L^−1^; Sanderson, Coe, et al. 2009). In addition, slope appeared to have no effect on δ^15^N patterns at salmon streams, whereas δ^15^N of non‐salmon riparian taxa tended to decrease slightly with stream slope, which suggests that if denitrification potential explains stream variation in δ^15^N, this is probably only at the non‐salmon sites. Alternatively, *Formica* spp. and *Pardosa* spp. may feed at a higher trophic level closer to the stream than at 100 m away, but that would not explain trends observed in the *Carex* spp. Denitrification, therefore, may be the landscape factor responsible for some of the enrichment in ^15^N of riparian plants and invertebrates, but the effect of stream type on δ^15^N was notable, suggesting that salmon presence also influences isotopic patterns in these riparian food webs.

### 4.2 C:N ratios and invertebrate body condition

Salmon organic matter can alter the riparian soil nitrogen pool (Gende et al., 2007), but the patterns we found in C:N ratios of *Carex* spp. were not consistent with a salmon‐mediated change in soil nitrogen. The *Carex* C:N ratios in our study were not different in salmon and non‐salmon streams. This contrasts with Helfield and Naiman (2001) who found that southeast Alaskan riparian trees had lower C:N ratios adjacent to streams with salmon than those without salmon spawners. However, *Carex* spp. have shorter life‐history strategies than trees and therefore are more likely to reflect recent patterns of SDN. Meanwhile, spawner abundances in southeast Alaska are several orders of magnitude higher than in Idaho (Heard, Shevlyakov, Zikunova, & McNicol, 2007). For example, Helfield and Naiman (2001) estimated that their streams received 3,000–8,000 kg of salmon‐derived nitrogen that, given a stream length of about 13 km, translates into about 200–600 kg of nitrogen per km. In comparison, using redd counts from 2009 in our study, streams ranged from 2.5 to 7 redds per km (Idaho Department of Fish and Game), and assuming (1) two adult fish per spawning redd, (2) an average Chinook biomass of 4.1 kg (excluding Alaska and British Columbia; Gresh, Lichatowich, & Schoonmaker, 2000), and (3) 3% of salmon biomass is nitrogen (Larkin & Slaney, 1997), the amount of salmon‐derived nitrogen ranges from 0.6 to 1.7 kg per km. If we estimate a salmon stream's total nitrogen budget from total nitrogen concentrations (260–290 μg N L^−1^) and discharge measurements (Table 1), we find that the contribution to each stream's nitrogen budget from salmon carcasses is likely <0.01%. The salmon‐derived nitrogen received in Idaho, therefore, is considerably lower than Alaska and other coastal areas. However, understanding the effect that salmon migrating over long distances have on organisms in inland streams is still important to establish, especially given the status of salmon within their native range.

Given the miniscule amount of salmon‐derived nitrogen currently being delivered to Idaho ecosystems, it should not be surprising that invertebrates adjacent to salmon streams did not appear to have lower invertebrate C:N ratios or enhanced body condition. However, understanding the benefits of contemporary SDN may be complicated by a historical effect of salmon, whereby enrichment in riparian taxa ^15^N reflects the legacy of past salmon runs instead of the presence of current spawners (Chambers, Marshall, & Danehy, 2004; Koyama, Kavanagh, & Robinson, 2005). For example, salmon runs in central Idaho have been declining for at least 50 years (Hassemer, Kiefer, & Petrosky, 1997; Isaak, Thurow, Rieman, & Dunham, 2003), and the amount of salmon‐derived nitrogen delivered to these streams in the 1950s would have been at least 10–20 kg per km. Even this modest amount of nitrogen compared to Alaska would still have been crucial to these oligotrophic ecosystems, which are severely nutrient‐limited (Naiman et al., 2002; Sanderson, Coe, et al. 2009). Thus, despite the 90% reduction in the amount of nitrogen brought in by salmon, the importance of SDN extends beyond its magnitude to the environmental context of the recipient ecosystem.

### 4.3 Percentage of salmon‐derived nitrogen incorporated

Salmon‐derived nitrogen appeared to have no effect on spider C:N ratio or body condition, but we did observe a weak trend in ants with higher C:N ratios associated with more salmon‐derived nitrogen. Lower C:N ratios can indicate greater quality of food for the next trophic level (Elser et al., 2000), but a higher C:N ratio for the organism as an individual could indicate greater lipid content (Logan et al., 2008; Post et al., 2007). In our study, the C:N ratio and δ^13^C were negatively correlated for ants. Because lipids tend to be depleted in ^13^C (Logan et al., 2008), the relationship between salmon‐derived nitrogen estimates and C:N ratios in ants could indicate that SDN can increase a consumer's lipid content; nevertheless, this relationship was rather weak (<10% of the variation was explained).

Contemporary inputs of salmon do not currently increase the quality of riparian plants or the body condition of riparian invertebrates in these ecosystems, which suggests that stable isotope patterns may reflect historical inputs of salmon. A historical legacy effect of SDN from salmon runs in prior years (Chambers et al., 2004; Koyama et al., 2005) could explain why δ^15^N of *Carex*,* Formica*, and *Pardosa* was generally higher at salmon streams than at non‐salmon streams. The retention within the watershed of past SDN has been suggested by the elevated δ^15^N conifer foliage observed near Idaho streams that historically bore salmon (Chambers et al., 2004; Koyama et al., 2005). The nature of this salmon legacy effect may also mean that short‐lived plants and invertebrates are unlikely to benefit from these sources as any putative SDN are bound up in long‐lived or persistent organic material, such as trees. In addition, even if the δ^15^N of riparian plants and invertebrates reflects the current availability of SDN, the average percentages in this study were still small (8%–10%) in comparison with the 20% percent observed in other regions with more abundant salmon populations, such as Alaska and western Washington (Bilby et al., 2003; Helfield & Naiman, 2001). This historical legacy effect likely explains not only some of the δ^15^N patterns in this study, but also those observed in other studies with declining salmon runs. Conflating the influence of past enrichment with that of current enrichment may be commonplace in the SDN literature, thus demonstrating that stable isotopes must be paired with physiological metrics to avoid errors in interpretation.

## 5 Conclusions

In contrast to riparian plants, no other published study has attempted to explicitly link SDN to the condition of riparian invertebrates. Therefore, very little is understood about the consequences of the nutrients that salmon bring to these terrestrial ecosystems for some of the most predominant consumers. Our study was unable to demonstrate any strong positive relationships between salmon nutrients and quality or body condition metrics, likely due to a historical salmon effect (Chambers et al., 2004; Koyama et al., 2005). Estimates of salmon carcass biomass in riparian ecosystems suggest that the amount of material can be substantial in places, with important consequences as others have posited (Bilby et al., 2003; Helfield & Naiman, 2001). However, the use of stable isotope ratios to infer that salmon nutrients are incorporated into riparian food webs does not directly assess the outcome of salmon impacts, unless the condition (e.g., body condition index or lipid content) or fitness (e.g., survival rates or clutch sizes) of recipient organisms is also quantified. To tease out the effects of biogeochemical and physiological processes as well as historical legacies of nitrogen sources, future ecological studies employing stable isotope analyses should be conducted along with field manipulations (e.g., carcass addition) and parallel laboratory experiments (e.g., feeding trials). Critically, SDN studies should pair the ecological distribution of stable isotope ratios with the physiological metrics of organisms.

## Conflict of interest

The authors declare that they have no conflict of interest.

## Data availability

Data are freely accessible at https://knb.ecoinformatics.org/#view/, doi:10.5063/F1SF2T4J

## Ethical approval

This article does not contain any studies with human participants or animals performed by any of the authors.
